# Apparent Diffusion Coefficient (ADC) Value: A Potential Imaging Biomarker That Reflects the Biological Features of Rectal Cancer

**DOI:** 10.1371/journal.pone.0109371

**Published:** 2014-10-10

**Authors:** Yiqun Sun, Tong Tong, Sanjun Cai, Rui Bi, Chao Xin, Yajia Gu

**Affiliations:** 1 Department of Radiology, Fudan University Shanghai Cancer Center; Department of Oncology, Shanghai Medical College, Fudan University, Shanghai, China; 2 Department of Colorectal Surgery, Fudan University Shanghai Cancer Center; Department of Oncology, Shanghai Medical College, Fudan University, Shanghai, China; 3 Department of Pathology, Fudan University Shanghai Cancer Center; Department of Oncology, Shanghai Medical College, Fudan University, Shanghai, China; The University of Tokyo, Japan

## Abstract

**Objective:**

We elected to analyze the correlation between the pre-treatment apparent diffusion coefficient (ADC) and the clinical, histological, and immunohistochemical status of rectal cancers.

**Materials and Methods:**

Forty-nine rectal cancer patients who received surgical resection without neoadjuvant therapy were selected that underwent primary MRI and diffusion-weighted imaging (DWI). Tumor ADC values were determined and analyzed to identify any correlations between these values and pre-treatment CEA or CA19-9 levels, and/or the histological and immunohistochemical properties of the tumor.

**Results:**

Inter-observer agreement of confidence levels from two separate observers was suitable for ADC measurement (k  =  0.775). The pre-treatment ADC values of different T stage tumors were not equal (*p*  =  0.003). The overall trend was that higher T stage values correlated with lower ADC values. ADC values were also significantly lower for the following conditions: tumors with the presence of extranodal tumor deposits (*p*  =  0.006) and tumors with CA19-9 levels ≥ 35 g/ml (*p*  =  0.006). There was a negative correlation between Ki-67 LI and the ADC value (r  =  −0.318, *p*  =  0.026) and between the AgNOR count and the ADC value (r  =  −0.310, *p*  =  0.030).

**Conclusion:**

Significant correlations were found between the pre-treatment ADC values and T stage, extranodal tumor deposits, CA19-9 levels, Ki-67 LI, and AgNOR counts in our study. Lower ADC values were associated with more aggressive tumor behavior. Therefore, the ADC value may represent a useful biomarker for assessing the biological features and possible relationship to the status of identified rectal cancers.

## Introduction

In recent years, colorectal cancer has become one of the leading causes of cancer-related death, while rectal cancer alone accounts for 30–35% of these cases [1]. The prognosis of rectal cancer depends on a number of factors, some of which are evaluated by the histopathology of collected surgical specimens/biopsies [2]. The impact of T and N stage rectal cancers on patient survival has been reported for pools of data collected from multiple randomized trials in which patients underwent surgery alone or surgery with some form of adjuvant treatment [3]. Some studies have also shown that the pre-treatment levels of carcinoembryonic antigen (CEA) and histological factors, such as tumor differentiation grade and the presence or absence of lymphatic vessel invasion are related to the overall prognosis [4]–[6]. The National Comprehensive Cancer Network (NCCN) supports these findings and regards T and N stages, peritumor-intravascular cancer emboli, extranodal tumor deposits, neural invasion, circumferential resection margins (CRM), and CEA levels as powerful prognostic factors [7].

Information on the microscopic structure of tumor tissues in addition to measurements of cell density, such as apoptosis and proliferation indexes, would be useful for cancer prognosis. Cell proliferation rates are likely indicative of the aggressiveness of specific tumors. The Ki-67 labeling index (LI), an established maker of cell proliferation, has traditionally been an optional immunohistochemical marker for the evaluation of the invasiveness and prognosis of rectal cancers [8]. Other immunohistochemical markers with established prognostic value include the following: 1) p53 and p21, which govern cell cycle checkpoints and apoptosis [9]–[10], 2) expression of CD44, which identifies cells that have lost their adhesion ability and/or have strong metastatic tendencies [11], 3) Her/neu, which influences cellular proliferation [12], and 4) the amount of AgNOR in a cell nucleus, which reliably reflects cell kinetics and can be used to assess the cellular proliferation rate; AgNOR counts increase with chronic inflammation, dysplasia, and malignancy [13]. These prognostic markers are valuable in the evaluation of tumor specimens obtained at the time of surgical intervention. However, a method that will enable the pre-operative assessment of cancer with the eventual aim of better assessing prognosis has been long awaited and could be used to tailor individual patient treatment to better combat disease.

Diffusion-weighted magnetic resonance imaging (DW-MRI), a modality of functional MRI, can be used to assess the biological characteristics of a tissue, including cellularity and water content [14]. The apparent diffusion coefficient (ADC) of water can be quantified from DWI images. This quantitative imaging biomarker has been shown to be useful in distinguishing benign from malignant lesions, while reflecting some of the histological characteristics of the lesion as well [14]–[15]. Several studies have shown that ADC values likely reflect the immunohistochemical features of a specific tumor and could then more precisely predict the aggressiveness and potential response of a particular tumor prior to initializing treatment [16]–[17].

Based on the aforementioned findings, we hypothesized that pre-treatment ADC values also could be used to identify specific biological features of rectal cancer and potentially predict tumor behavior. The aim of this study was to investigate the possible correlation between ADC values and the clinical, pathological, and immunohistochemical features of selected patients/tumors to evaluate the efficacy of using pre-treatment tumor ADC values to predict the behavior of and treat rectal cancers.

## Materials and Methods

### Patients

Between January and December 2012, 49 patients, which were diagnosed and treated at the Fudan Cancer Hospital, were selected as subjects in this retrospective study. Selection criteria included the following: 1) a histological biopsy of proven rectal carcinoma, 2) treatment by surgical resection without neoadjuvant therapy, 3) the availability of pathological reports of surgical specimens that referred to the tumor differentiation grade, and 4) evaluation via MRI with DWI. Our initial analysis identified 56 patients that matched the criteria above. Exclusion criteria included the following: 1) a long interval between MRI and surgery greater than three weeks (3 patients excluded), 2) no identified tumor signal on a DWI and ADC map (2 patients excluded), and 3) a motion artifact apparent on the DWI (2 patients excluded). Clinical and imaging data were retrieved from the patient database. The study data were retrieved from a previous imaging study, and we obtained essential approval from the local institutional ethical committee; informed consent was obtained from all patients included in the study. The final study population consisted of 49 remaining patients (28 males and 21 females). The median study age was 59 years with a range of 28–84 years. Thirty patients received Dixon surgery, while 15 patients underwent Miles surgery, and 4 patients received Hartmann surgery.

### MR Imaging

Magnetic resonance imaging was performed using a 3.0 Tesla (T) MR magnet (Signa Horizon, GE Medical Systems, Milwaukee, WI) with a phased-array body coil. The standard imaging protocol consisted of sagittal T2-weighted (T2W) fast spin echo and oblique axial thin-section T2W, which were used to assess T staging (repetition time/echo time [TR/TE]: 3420/110 ms, flip angle: 90°, echo train length: 16, field of view [FOV]: 20 cm, section thickness: 3 mm, interspace: 0.5 mm, number of slices: 20 slices, and acquisition time: 6 min 25 s). Axial diffusion-weighted images (DWI) were obtained using the following parameters: b-values: 0, 800 s/mm^2^, TR/TE: 2800/67 ms, echo planar imaging [EPI] factor: 53, number of slices: 28 slices, and acquisition time: 2 min 30 s. Enhanced images were acquired after the intravenous administration of gadofosveset trisodium using axial LAVA sequence (TR/TE: 3.4/1.5 ms, flip angle: 15°, FOV: 33 cm, section thickness: 4.8 mm, interspace: 0 mm, number of slices: 38, acquisition time: 19 s). Patients did not receive bowel preparation antispasmodic medication or rectal distention before MR examinations.

### ADC Evaluation

The MRI images were independently reviewed on a picture archiving and communication system (PACS) by two gastrointestinal radiologists (Y.J.G and T.T). The two gastrointestinal radiologists were blinded to information obtained at surgery and pathological analysis. To avoid any recall bias, the order of cases was changed in each review session. One professor had more than 25 years experience, while the less experienced professor had 5 years of experience in interpreting abdominal MRI images. ADC maps were calculated from DWI by using Functool software at a GE AW 4.3 workstation (GE Healthcare, Wisconsin, Waukesha, USA). ADC measurement was calculated from a sample of three regions of interest (ROIs) that were drawn manually within the solid tumor parts of three independent tumor-containing slices by the radiologists. The size and position of the ROIs was selected to cover the entire tumor area on a single section containing the largest available tumor area.

### Potential Prognostic Factors

#### Histological diagnosis

Clinical and histological prognostic factors were obtained from the clinical patient database to investigate the association between ADC values and potential prognostic factors. Histological evaluation of the surgical resection specimens was the reference standard for the histological parameters. Pathologic reports were reviewed to determine the tumor T stage, N stage, histological type, differentiation grade, peritumor-intravascular cancer emboli, extranodal tumor deposits, neural invasion, and CRM. Pathological TNM stage was determined according to the 2007 TNM system [18]. The clinical factors examined were the plasmatic CEA (ng/ml) and CA19-9 (U/ml) levels at the time of diagnosis.

### Immunohistochemistry

The investigation was comprised of immunohistochemical analyses of tumor tissues from surgical intervention. Immunohistochemical staining was performed for Ki-67, p21, p53, CD44, Her/neu, and AgNOR according to manufacturer's instructions (Shanghai Roche Pharmaceuticals Ltd., Long Island, NY, USA). The final values of Ki-67 LI were represented as the percentage of positive Ki-67 labeled cells identified when counting a total of at least 1000 neoplastic nuclei, subdivided into 10 fields with obvious staining were chosen and examined at 400× magnification. Positive Ki-67 labeled cells were defined as the presence of a distinct brown staining in the cytoplasm of neoplastic cells [16]. Marker expression analysis was subdivided into two categories: positive and negative staining for p21, p53, CD44, and Her/neu. The mean number of AgNOR dots per cell was calculated after silver nitrate staining; this value was set as the AgNOR count. The procedure was performed by an experienced pathologist who had no access to patient data or any information regarding the clinical patient status.

### Statistical Analysis

Statistical analyses were performed using the Statistical Package for Social Sciences (SPSS, version 13.0, Chicago, IL, USA). All *p* values <0.05 were considered statistically significant. ADC value, Ki-67 LI, and AgNOR count were reported as the mean ± standard deviation. The threshold of CEA and CA19-9 levels was used in our institution. For statistical analysis, Student t-tests (independent-samples t-test) were used to assess the differences between means of the following groups: the presence versus absence of peritumor-intravascular cancer emboli, the presence versus absence of extranodal tumor deposits, the presence versus absence of neural invasion, the presence versus absence of CRM, CEA <5.2 ng/ml versus ≥ 5.2 ng/ml, CA19-9 <35.5 U/ml versus ≥ 35.5 U/ml (threshold used in our institution), the presence versus absence of p21, the presence versus absence of p53, the presence versus absence of CD44, and the presence versus absence of Her/neu. The Kruskal-Wallis test was used to assess differences between the means of three different histological types, three differentiation grade groups, four different T stages, and three different N stages. Correlations between Ki-67 LI and ADC values and correlations between AgNOR counts and ADC values were investigated with the determination of the Spearman's correlation coefficient followed by a One-Sample Kolmogorov-Smirnov Test.

To evaluate inter-observer agreement regarding the measurement of tumor ADC values, k statistics were used. A k value of less than 0.20 indicated poor agreement, a k value of 0.21–0.40, fair agreement, a k value of 0.41–0.60, moderate agreement, a k value of 0.6–0.80, good agreement, and a k value of more than 0.81, excellent agreement.

## Results

### Clinical and Histopathological Findings

The tumor characteristics of 49 rectal cancer patients are listed in Tables 1 and 2. The 49 patients in our study exhibited varying types of rectal cancer as follows: 44 patients had pure adenocarcinomas, 2 patients had signet-ring carcinomas, and 3 patients had mucinous adenocarcinomas. Of these, 8 patients were poorly differentiated, 37 were moderately differentiated, and 4 were well differentiated. Four patients were staged as T1, 23 as T2, 19 as T3, and 3 as T4. Thirteen patients were staged as N0, 12 as N1, and 24 as N2. In 14 patients, peritumor-intravascular cancer emboli were absent, while they were present 35 patients. In 11 patients, extranodal tumor deposits were absent, while they were present in 38 patients. In 40 patients, neural invasion was absent, while it was present in 9 patients. In 46 patients, CRM were absent, while they were present in 3 patients. At the time of diagnosis, 26 patient CEA levels were lower than 5.0 ng/ml, while the remaining 23 patient levels were equal to or greater than 5.0 ng/ml. At the same time, 8 patients had CA19-9 levels lower than 35 U/ml, and 41 patients had CA19-9 levels equal to or greater than 35 U/ml.

**Table 1:** Correlation between Histological, Clinical Parameters, and ADC values.

Parameters	N(%)	ADC values (Mean ± SD) × 10^−3^ mm^2^/s	P value
**Histology** a			0.123
Signet-ring carcinoma	2	1.15 ± 0.08	
Adenocarcinoma	44	1.30 ± 0.21	
Mucinous adenocarcinoma	3	1.40 ± 0.22	
**Histological grade** a			0.515
Poorly differentiated	8	1.20 ± 0.24	
Moderately differentiated	37	1.31 ± 0.23	
Well differentiated	4	1.32 ± 0.14	
**T category** a			**0.003**
T1	4	1.53 ± 0.29	
T2	23	1.38 ± 0.16	
T3	19	1.22 ± 0.27	
T4	3	1.19 ± 0.19	
**N category** a			**0.055**
N0	13	1.42 ± 0.21	
N1	12	1.32 ± 0.17	
N2	24	1.22 ± 0.21	
**Peritumor-intravascular cancer emboli** *			0.061
Negative	14	1.37 ± 0.23	
Positive	35	1.25 ± 0.18	
**Extranodal tumor deposits** *			**0.006**
Negative	11	1.43 ± 0.16	
Positive	38	1.25 ± 0.19	
**Neural invasion** *			0.890
Negative	40	1.28 ± 0.21	
Positive	9	1.29 ± 0.13	
**CRM** *			0.312
Negative	46	1.31 ± 0.22	
Positive	3	1.18 ± 0.13	
**CEA** *			0.691
<5 ng/ml	26	1.31± 0.20	
≥ 5 ng/ml	23	1.29 ± 0.23	
**CA19-9** *			**0.006**
<35 g/ml	8	1.49 ± 0.26	
≥ 35 g/ml	41	1.26 ± 0.19	

*Independent-samples t-test.

a The Kruskal-Wallis test.

CRM means circumferential resection margin.

**Table 2:** Correlations between Immunohistochemical Parameters and ADC values.

Parameters		N	ADC value (Mean ± SD) x 10^−3^ mm^2^/s	P value
				r
P21*	(−)	30	1.32 ± 0.25	0.756
	(+)	19	1.30 ± 0.20	–
P53*	(−)	35	1.31 ± 0.23	0.776
	(+)	14	1.29 ± 0.19	–
Her2/neu*	(−)	44	1.29 ± 0.16	0.976
	(+)	5	1.28 ± 0.07	–
CD44*	(−)	11	1.32 ± 0.22	0.251
	(+)	38	1.23 ± 0.20	–
Ki-67*	68.97 ± 16.98 %	49	1.30 ± 0.21	0.026
				r = −0.318
AgNOR*	2.96 ± 0.84	49	1.30 ± 0.21	0.030
				r = −0.310

*Independent-samples t-test.

*Spearman's correlation analysis.

### Immunohistochemical Findings

Table 2 lists the immunohistochemical information of all 49 patients. Of these, 30, 35, and 11 patients lacked expression of p21, p53, and CD44, respectively. Therefore, 19, 14, and 38 patients had positive expression of p21, p53, and CD44, respectively. Her/neu expression was negative in 44 patients (positive in 5). The mean of Ki-67 LI for all patients was 68.97 ± 16.98 % and the AgNOR count was 2.96 ± 0.84.

### Correlation between ADC value and Prognostic Factors (Histological and Clinical Parameters)

**Figure 1:**
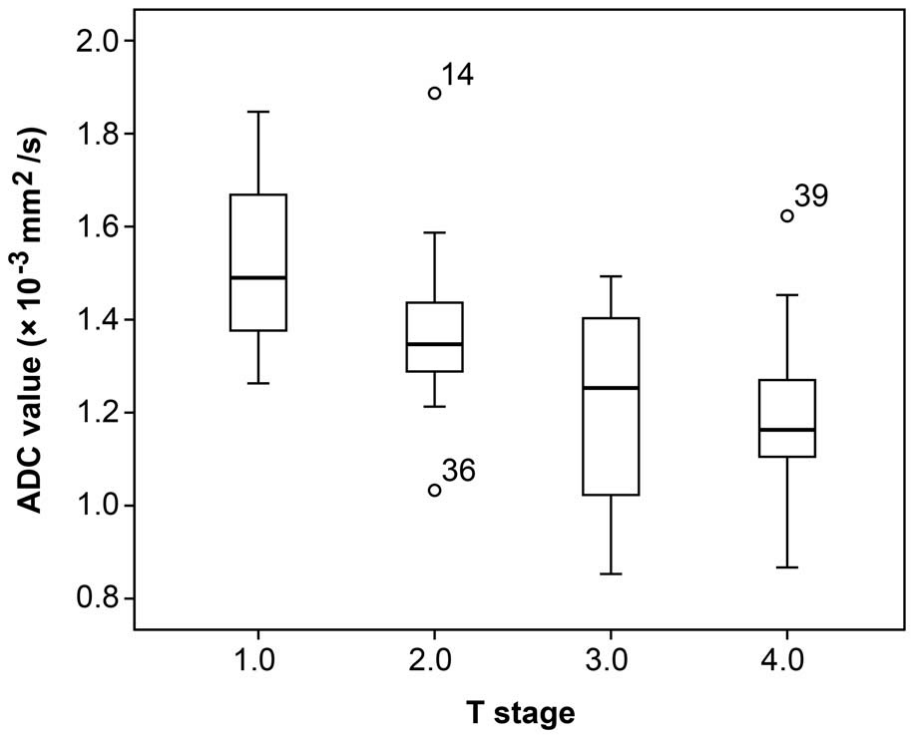
Comparison of mean AD values of tumors according to the T stage. The whiskers represent the standard deviation.

Inter-observer agreement of confidence levels for observers 1 and 2 was adequate for ADC measurement (k  =  0.775). The mean ADC value for all patients was 1.30 ± 0.21 × 10^−3^ mm^2^/s. The difference in ADC values between different groups and their associations with various histological and clinical parameters are outlined in Table 1. Tumor T stage, extranodal tumor deposits, and CA19-9 levels were significantly associated with ADC values. The ADC values of different T stage tumors were not equal (*p*  =  0.003); the overall trend indicated that the higher the T stage, the lower the ADC value. The relationship between ADC values and T stage are shown in Figure 1. ADC values were significantly lower under the following conditions: 1) tumors with the presence of extranodal tumor deposits (*p*  =  0.006) and 2) tumors with CA19-9 levels ≥ 35 g/ml (*p*  =  0.006). The N stage also increased as the ADC value decreased, although this trend was not statistically significant. In addition, the patients with peritumor-intravascular cancer emboli, neural invasion, and CRM and CEA levels ≥ 5 ng/ml had lower ADC values. This trend was also not statistically significant.

### Correlation between ADC value and Prognostic Factors (Immunohistochemical Parameters)

**Figure 2:**
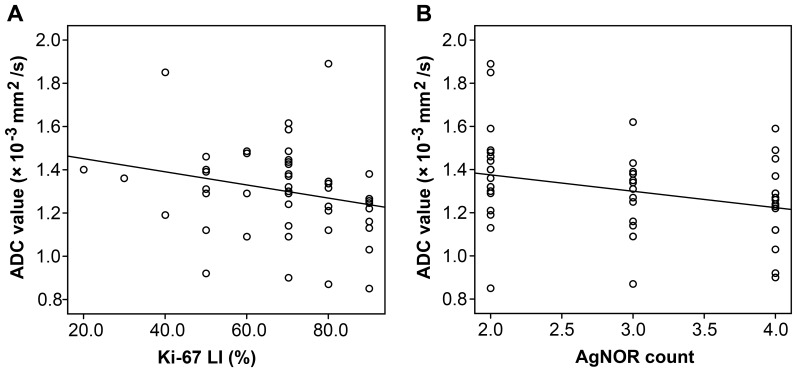
The relationships between ADC values and Ki-67 LI (A) and ADC values and AgNOR count (B).

**Figure 3:**
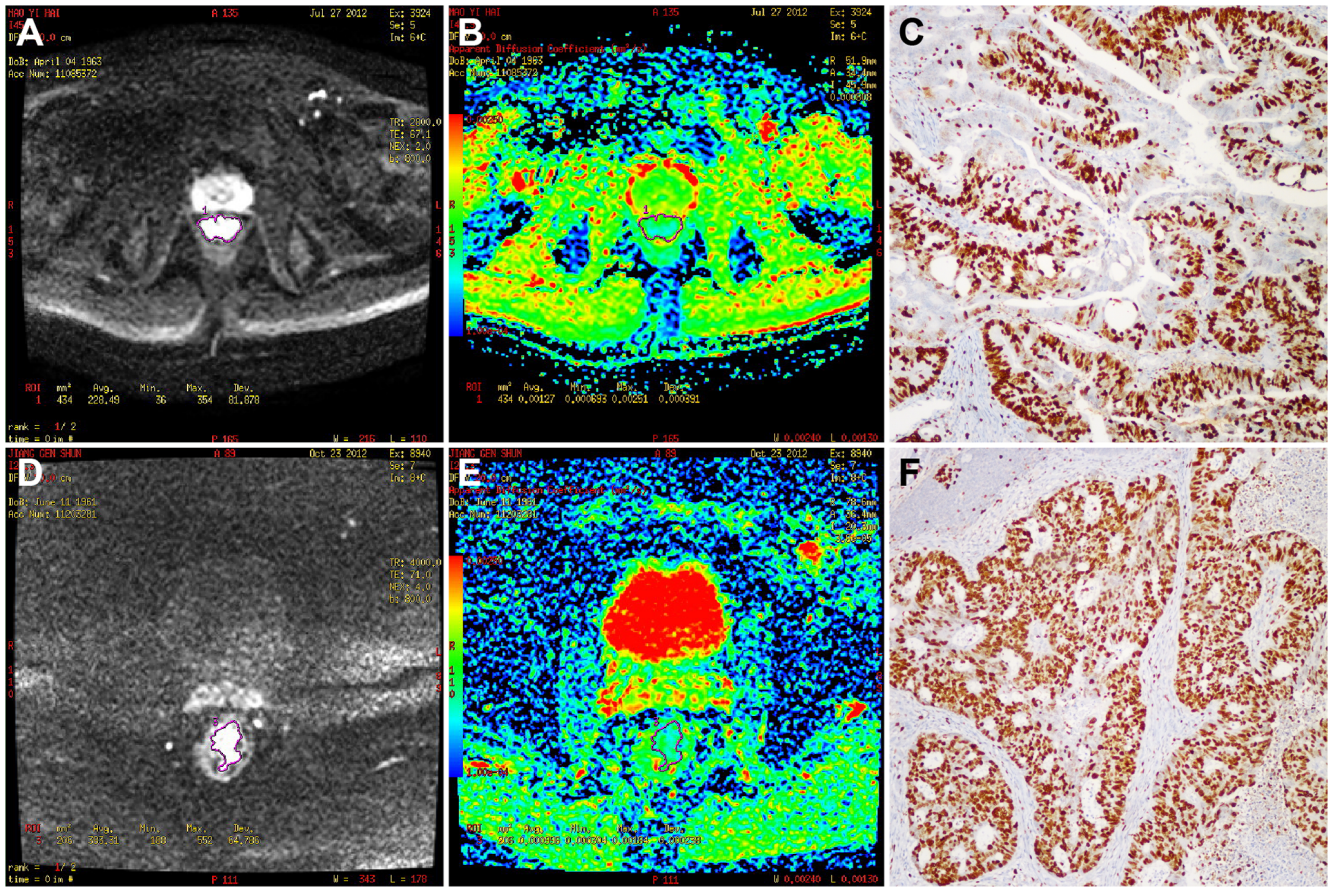
ADC values were measured in tumors with different Ki-67 LI values. In a low Ki-67 LI (Ki-67 value  =  50% and AgNOR count  =  2), which was limited to the bowel wall, without mesorectal lymph nodes, the ACD value (1.27 × 10^−3^ mm^2^/s, ROIs  =  434 mm^2^) was higher than in a high Ki-67 LI (A: b  =  800 s/mm^2^c image, B: ADC map, C: Ki-67 Immunohistochemical staining). In the high Ki-67 LI range (Ki-67 LI  =  90% and AgNOR count  =  4), in a tumor staged as T3N2, the ADC value (1.00 × 10^−3^ mm^2^/s, ROIs  =  438.3 mm^2^) was lower (D: b  =  800 s/mm^2^c image, E: ADC map, F: Ki-67 Immunohistochemical staining).

Table 2 shows the differences in ADC values among different groups of immunohistochemical parameters. The group positive for p21, p53, CD44, and Her/neu, had lower ADC values, although these differences were not statistically significant. Ki-67 LI (r  =  −0.318, *p*  =  0.026) and AgNOR counts (r  =  −0.310, *p*  =  0.030) were negatively correlated with ADC values by Spearman's correlation analysis. The relationships between ADC values and Ki-67 LI and ADC values and AgNOR counts are displayed in Figures 2 and 3.

## Discussion

Several studies have shown that new MRI techniques may provide both morphological and functional parameters that can be correlated with measurements of tumor biology [19]–[20]. For this reason, new MRI techniques could have the potential to identify, quantify, and assess new/improved cancer biomarkers. The goal of the present work was to assess the value of using DWI as a potential noninvasive imaging technique to evaluate the biological features of rectal cancer tumors. Our results show that lower ADC values are strongly associated with higher T stages, an elevated Ki-67 LI, elevated AgNOR counts, the present of extranodal tumor deposits, and CA19-9 levels that are below a 35 g/ml threshold. Although the rest of subgroups do not show statistically significant correlations, there were trends suggesting that lower ADC values may also be associated with a more aggressive tumor profile. These results complement those of a recent study, which suggested that tumor ADC values may be used to predict the behavior of certain rectal cancers [2].

The NCCN has previously stated that T stage, N stage, extranodal tumor deposits, and CEA levels are powerful prognostic factors for predicting the overall disease-free survival of rectal cancer patients [7]. We found that pre-treatment ADC values were significantly lower for tumors with higher T stages, extranodal tumor deposits, and CA19-9 levels below 35 g/ml. Therefore, we believe that ADC values may indeed be a powerful prognostic indicator during the assessment and treatment of rectal cancer. This may be explained by the fact that ADC values are derived from the diffusive movement of water molecules, which is often influenced by microstructure, cell density, and other histological components present in the tissue at a microenvironmental level. Therefore, ADC levels might reflect the aggressiveness of a particular tumor tissue. Tumor differentiation grade has also been identified as an important prognostic factor; the results of a study by Curvo-Semedo et al. [2] demonstrated that there was a statistically significant correlation between ADC value and tumor differentiation grade upon histological examination. However, there was no statistically significant correlation between ADC value and tumor differentiation grade in our study. This may be attributed to the fact that the ROIs we drew over the tumor were as large as possible when the Curvo-Semedo study selected smaller round/oval-shaped ROIs. These differences in measuring techniques would most likely result in different outcomes. The NCCN has also stated that CEA level may be another important prognostic factor [7]. However, our results showed no correlation between ADC values and CEA levels. Therefore, we suggest that the CEA level at the time of diagnosis might not reflect the actual status of the disease.

NCCN risk grouping (as mentioned above) may be clinically helpful. Integration of immunohistochemical features into risk stratification schemes could also be useful in clinical practices [21]. In this study, we also compared ADC values in various tumor subgroups defined by combinations of immunohistochemical features that offered important information regarding tumor apoptosis and proliferation during antitumor therapy. Proliferation measurements showed an increase in mitotic activity, hyperchromatism, and the N/C ratio, which would theoretically decrease water diffusivity in the intracellular tumor space, contributing to a reduced ADC value [22]. The Ki-67 LI, AgNOR count, p53, p21, CD44 and Her/neu all play important roles in apoptosis and/or tumor proliferation related to prognosis. Several studies had shown that there is negative correlation between ADC value and the Ki-67 LI in bladder cancer and neuroepithelial tumors [8], [23]. In addition, one study showed that low ADC values were related to the positive expression of estrogen receptor (ER) (*p*  =  0.027) and the negative expression of human epidermal growth factor 2 (HER2) (*p*  =  0.018) [24]. In our study, we also found that ADC values had a negative correlation with the Ki-67 LI and AgNOR count (*p*  =  0.026 and 0.030, respectively). There was also a trend that lower ADC values were associated with a more aggressive tumor profile, although the rest of immunohistochemical parameters did not show any statistically significant correlations. Ultimately, we can conclude that the ADC value may be a potentially powerful imaging biomarker, although additional studies are necessary.

Nonetheless, there were some limitations in our study. First, biases could be present in our cohort, because most of patients presenting with locally advanced rectal cancers underwent neoadjuvant therapy, limiting our study population. Second, the ADC values were obtained on three sample ROIs containing the largest available tumor, which might not be fully representative of the overall tumor profile. However, this approach was chosen, because outlining the whole tumor volume was time-consuming and difficult to perform in clinical practice. Third, we used the final histological T stage, N stage, and other factors for correlation with ADC values derived from the primary MRI, in which these factors were no longer representative of the initial tumor profile. Fourth, the number of patients who met the criteria was relatively small due to our exclusion strategy. We suggest that prognostic significance of ADC values should be investigated in prospective studies with a much larger patient cohort and a longer follow-up period, which was beyond the scope of the current study.

In conclusion, this study indicates that the pre-treatment ADC values of rectal cancer can be correlated with important biological features of tumors, which may bear prognostic significance. Pre-treatment ADC values show potential as a new noninvasive imaging biomarker that may be helpful in predicting the biological properties of rectal cancers.

## Supporting Information

Data S1 **Data for analysis of this article.**
(XLS)
